# Environmentally Optimal, Nutritionally Sound, Protein and Energy Conserving Plant Based Alternatives to U.S. Meat

**DOI:** 10.1038/s41598-019-46590-1

**Published:** 2019-08-08

**Authors:** Gidon Eshel, Paul Stainier, Alon Shepon, Akshay Swaminathan

**Affiliations:** 1 Physics Department, Bard College, Annandale-on-Hudson, NY 12504-5000 USA; 2 Harvard College, Cambridge, MA USA; 3 Department of Nutrition, T. H. Chan School of Public Health, Harvard University, Boston, USA

**Keywords:** Element cycles, Environmental impact, Obesity

## Abstract

Because meat is more resource intensive than vegetal protein sources, replacing it with efficient plant alternatives is potentially desirable, provided these alternatives prove nutritionally sound. We show that protein conserving plant alternatives to meat that rigorously satisfy key nutritional constraints while minimizing cropland, nitrogen fertilizer (Nr) and water use and greenhouse gas (GHG) emissions exist, and could improve public health. We develop a new methodology for identifying nutritional constraints whose satisfaction by plant eaters is challenging, disproportionately shaping the optimal diets, singling out energy, mass, monounsaturated fatty acids, vitamins B_3,6,12_ and D, choline, zinc, and selenium. By replacing meat with the devised plant alternatives—dominated by tofu, soybeans, peanuts, and lentils—Americans can collectively eliminate pastureland use while saving 35–50% of their diet related needs for cropland, Nr, and GHG emission, but increase their diet related irrigation needs by 15%. While widely replacing meat with plants is logistically and culturally challenging, few competing options offer comparable multidimensional resource use reduction.

## Introduction

Agriculture is among the key ways humans impact^1^—mostly adversely^2^—natural environments. While livestock production contributes disproportionally to these impacts both per kilocalorie (kcal) and per gram (g) protein^3–5^, producing plant based items for direct human consumption is less resource intensive^6^. Plant-based alternatives to meat are thus potentially desirable^7^, provided they can be rigorously shown to quantitatively enjoy nutritional and environmental consequences that are at least benign, but preferably beneficial^8–11^. We have recently quantified the generalized losses associated with feed-to-food conversion in the U.S. food system and the potential environmental and food security benefits of eliminating these losses^12–14^. We have also identified the primary reason for these losses, large land use disparities between animal based products and their nutritionally equivalent plant based alternatives^13^, and quantified the resource use and nutritional outcomes expected from a nutritionally sound, protein equivalent plant replacements of beef in the U.S. diet^12,14^. Yet while beef is by far the most resource intensive^12–14^ poultry and pork also use more resource than most plant alternatives^15,16^, and replacing them is likely to further improve the environmental performance of food systems^17^.

Despite the above expectations, the nutritional and environmental consequences of replacing all meat in the mean U.S. diet with plant alternatives in a nutritionally rigorous manner have been only preliminarily explored. We thus employ linear programming to devise hundreds of plant based partial diets that replace beef alone or its sum with pork and poultry, the dominant U.S. meat types^18^. All minimize combined environmental costs (as described shortly and in the Methods section) while satisfying 44 nutritional constraints, but each comprises a distinct randomly chosen subset of available plant items (a randomization based solution strategy often called Monte Carlo, hereafter MC).

As in our earlier work cited above, here “replacing” means exact replacement of the protein content of the forgone meat (i.e., protein is an equality constraint) while satisfying 43 additional inequality constraints of both signs that collectively ensure the plant replacement diets are at least as nutritious as the meat they replace. The protein conservation does not imply the current protein intake is optimal, a claim we do not make. Rather, it is simply meant to facilitate meaningful “per g protein” nutritional and environmental comparison with the current situation. With the above definition of “replacement”, the first step of the calculation is quantifying the nutritional contributions of the replaced meat(s) to the mean American diet. We do that by combining mean 2000–2016 Department of Agriculture (USDA) consumption data^18^ and per g nutritional composition^19^. Plant based replacements must therefore supply the 12 + 4 + 14 ≈ 30 g protein d^−1^ Americans currently derive from beef, pork, and poultry respectively (out of corresponding approximate total masses of 70 + 30 + 74 g meat d^−1^), or the 12 g protein d^−1^ due to beef alone.

While here we consider as potential replacements only plant items, whose resource needs per g protein are mostly lower than even the resource efficient dairy and eggs^5,11,16,20^, it does not follow that pure vegetal diets are globally optimal when simultaneously optimizing nutritional and resource use objectives. For example, replacing pork with rice would reduce saturated fat intake but reduce greenhouse gas (GHG) emissions only minimally while nearly doubling water use. It is thus possible that when allowing livestock based items, seafood, or novel foods^21^ to compete, the optimization methodology may include them in some replacement diets. We plan to explore this possibility elsewhere, and here focus exclusively on purely plant based replacement diets.

Seeking nutritional–environmental harmony, here we augment our earlier approach^12,14^ in two methodologically important ways. First, by adding water use to the earlier land use, GHG emissions and reactive nitrogen (Nr) use, the resource use minimization takes note of four rather than three resources. Second, here we minimize the use of all four resources simultaneously (after standardizing and combining them, with weights reflecting the importance of meat production to the respective national total burdens, as described in the Methods section), while earlier^12^ we minimized each individually.

## Results and Discussion

### Nutrient delivery

**Figure 1:**
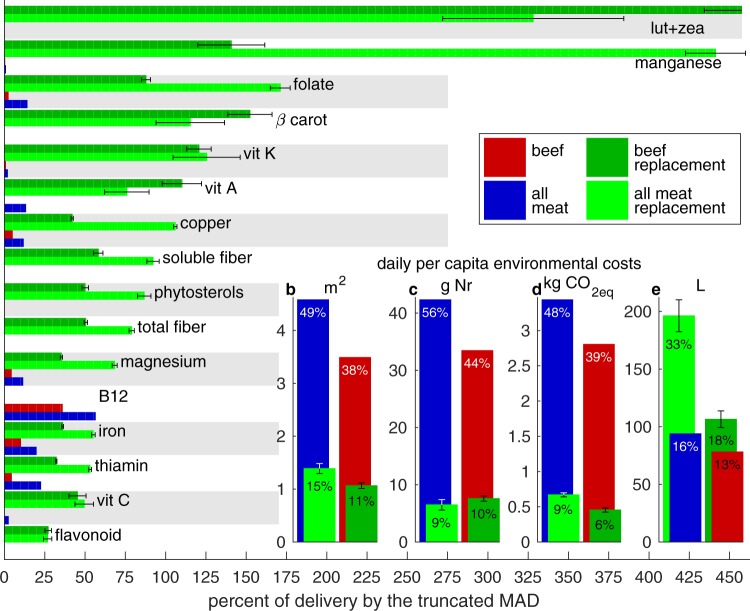
Key nutritional (main) and environmental (insets **b**–**e**) consequences of replacing all meat (blue) and beef only (red) in the mean American diet (MAD) with protein conserving nutritionally sound plant based alternative diets. Panel a presents mean ± 1 standard deviation nutrient deliveries by the 500 MC plant replacement diets (bar lengths and whiskers) for all nutritional attributes whose two delivery differences from the respective replaced meat both exceed 25% of the corresponding delivery by the truncated mean American diet (tMAD, the nutrient delivery by all 73 plant and animal food items we consider, more formally defined in the Methods section). Alternating gray shading helps distinguish individual attributes. Insets (**b**–**e**) show use of land and reactive nitrogen (Nr), greenhouse gas emissions, and irrigation needs by the meat diets and their plant based replacement (with color convention shown by legend). The percent of total per capita dietary use of the resource (associated with the truncated MAD) to which values correspond are indicated numerically.

Figure 1 (main) presents the 16 nutritional attributes whose delivery changes due to the meat to plant shifts exceed 25% of the approximate delivery by the full MAD (with further details in the Methods section).

Of these 16 leading nutritional additions (note that all 16 changes are positive), all save vitamin B_12_—of which the replaced meats are a solid source while the plant diets deliver none—are nutritionally protective and desirable^22,23^. Increasing consumption of them, which is expected to accompany the putative shift to the plant based replacement diets under the assumption of consumption = production, is thus expected to improve public health. The lost B_12_ delivery represents a well-known^24^ and potentially serious^25^ limitation of plant based diets, which fortunately can be readily alleviated by supplementation^26^. In terms of individual nutrient delivery, replacing all meat or beef with the proposed plant alternatives devised here is thus mostly advantageous^22,27^.

### Resource use consequences

The expected direct daily per capita resource use consequences of the dietary shifts are presented in Fig. 1b–e and fall into two categories. The first comprises considerable reductions in the need for cropland, Nr, and GHG emission, amounting to saving 35–50% of the total dietary use of the resources. Conversely, the replacement plant based diets require additional irrigation that amounts to 5–15% of total current dietary water needs.

**Figure 2:**
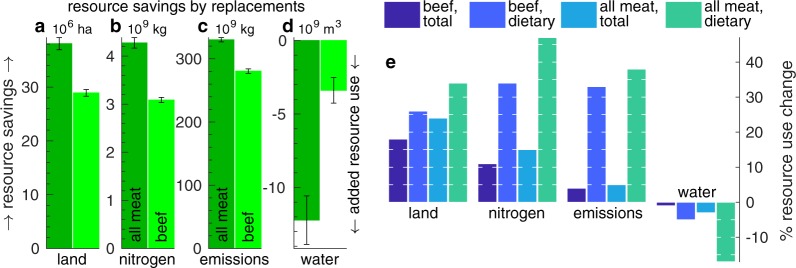
Absolute (**a**–**d**) and relative (**e**) changes in national resource use associated with a hypothetical full population deployment of the considered all meat or beef only replacements (left and right bars of each metric respectively in **a**–**d**). Since the shown values are savings, positive ones mean savings and negative ones mean increased usage (see arrows on left of **a** and right of **d**). Panel e shows the changes as percent of the annual national total and dietary (associated with producing the truncated MAD) using the shown colors. White tick marks are 5% apart. Replacing all meat with plant based alternatives, e.g., will save about 34% and 24% of the current national dietary and total land use (2 rightmost land bars in **e**).

Next, in Fig. 2 we estimate national level consequences of a hypothetical adaptation of the meat-to-plant dietary shifts by the full U.S. population as the direct impacts per person committing to the dietary shift (Fig. 1e) times 327 million Americans. This rests on several key assumptions ranging in robustness. First, we assume sparing of pastureland used for beef grazing, not reallocation to other modes of food production. This is robust, as while vanishing beef consumption obviates grazing, most range and other extensive grazing lands are ill suited for crop production^28^.

Second, we assume reallocation of high quality cropland currently used for feed production to production of the plant items that dominate the solution replacement diets with unchanged national mean yields. While surely not inevitable, this is consistent with evidence offered by current geographical variability and historical precedent for agronomic suitability of cropland to diverse crops. For example, the key protein source in the replacement diets, soy, is grown^29^ in widely diverse geographies from cool, continental North Dakota to maritime hot and humid North Carolina, with optimal location around Iowa. These States’ respective 2017 mean annual soybean yields^29^ were 35, 40 and 57 bu ac^−1^, about −29%, −19% and +16% above the 2017 national mean, 49.3 bu ac^−1^. The respective 1980 values were^29^ 17, 18 and 39 bu ac^−1^, about −34%, −32% and +45% above the 1980 national mean, 26.5 bu ac^−1^. Thus as the national mean rises with time due to improved cultivars and agricultural practices, geographical variability regresses to the mean, with yields in suboptimal states slowly approaching the rising national mean while optimal states enjoying decreasing edge with time. That is, technical adaptation erodes the importance of geographically variable suitability for particular crops.

The key shortcoming of the above approach to estimating national level resource savings is that it does not, and cannot, address a full restructuring of the U.S. food system in response to the shifting food demands. Addressing this restructuring requires a full agro-economic model of the U.S. food system, which is well beyond the scope of this paper. It is thus fair to consider the estimated national level resource saving we report here as an upper bound on actual expected savings.

With these stipulations in mind, these calculations yield two sets (for the all meat or beef only replacements), each comprising four resource use changes corresponding to the four considered resources. For example, we find (Fig. 2a–d) that replacing U.S. beef with plant alternatives stands to save annually approximately 29 (28, 30) million cropland ha, 3 (3.0, 3.1) billion kg Nr, 280 (276, 283) billion kg CO_2e_, and −3 (−5, −3) billion m^3^, with parenthetical values denoting ± one standard deviation about the mean.

To test the plausibility of the above estimates, we use a recent independent estimate^30^ of 48.4 kg CO_2e_ (kg boneless edible beef)^−1^. About 15% of these emissions occur post farm gate (their Table 2). Consistency with our emission data thus requires reducing this to 85%, which yields about 41 kg CO_2e_ (kg farm gate beef)^−1^. Multiplying this by the 8.1 billion kg annual national beef consumption (which is the annual equivalent of the ≈70 g d^−1^ daily per capita beef consumption introduced earlier times 327 million Americans) yields 333 billion kg CO_2e_ y^−1^ emissions due to beef production. Subtracting the 55 billion kg CO_2e_ y^−1^ emissions required for producing the plant replacement diet yields 278 billion kg CO_2e_ y^−1^ difference, within 1% of our 280 billion kg CO_2e_ y^−1^ estimate. The two estimates thus agree closely.

The full set of expected national resource changes is shown in Fig. 2. Because meat production accounts for 5–10% of the total national GHG emissions and fresh water use, the reduced GHG savings and added water consumption shown in Fig. 1d,e translate to national level consumption changes (after accounting for the resource needs of the replacement plant diets) of only −5 and +3% of the respective total national resource uses. Conversely, because feeding livestock requires 36 and 21% of the national cropland^31^ and reactive nitrogen application^5,20^, the dietary shifts offer considerable savings of these resources, ≈10–20% of the respective current total national use of these resources.

### Diet composition, nutrient delivery, and share of resource use

In daily per capita mass, tofu, soybeans, peanuts, and lentils dominate the all meat replacement, while green peas, lentils, asparagus, and spinach dominate the beef only replacement (Fig. S1a,b, which present the eight most dominant plant items in the mean replacement diets calculated over the 500 Monte Carlo realizations). Because these lists partly reflect the list of plant items we use and the upper mass bounds we impose (see Methods), they are unlikely to be the globally optimal plant replacements to the two meat masses and types (that is, more nutritious or environmentally sound alternatives may exist using items not included here). They also take no note of tastes, cuisines or palates, and may thus prove suboptimally deployable. Together, these two limitations of the presented solutions suggest that similarly (or more) environmentally and nutritionally desirable plant (or mixed) diets which better suit specific tastes and culinary preferences may exist.

**Figure 3:**
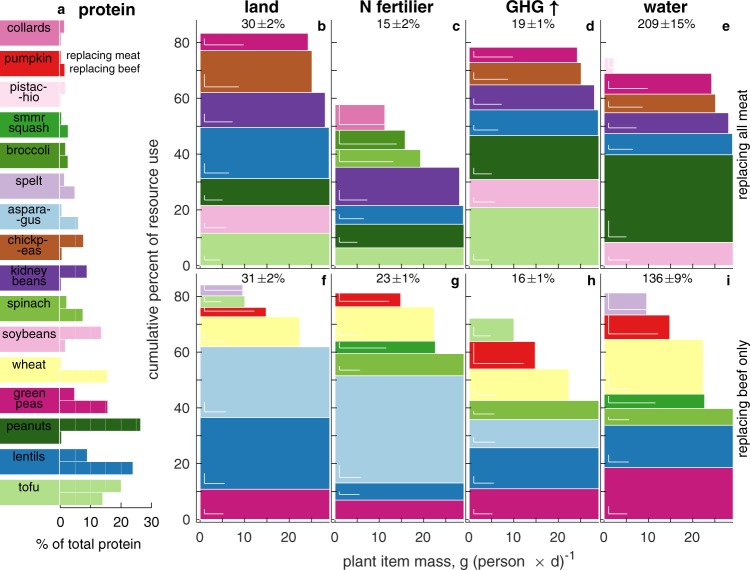
Key resource using items in the plant based all meat (**b**–**e**) and beef (**f**–**i**) replacement diets, ranked by mass contributions (horizontal axes). Nr ≡ reactive nitrogen fertilizer; GHG ≡ greenhouse gas emissions. Panel a: protein contributions of leading item to the right of the item legend identifier, with the upper (lower) bar corresponding to the all meat (beef only) replacements. Panels b-i: items’ mass and resource use contributions by the plant based replacement diets. Rectangles’ horizontal extents show items’ masses, with vertical extents showing corresponding resource uses. For example, contributing ≈29 g cap.^−1^ d^−1^, spinach is prominent (4^th^ by mass) in the beef replacement diet. Yet because it is not a top land user, it is thus absent from panel f. Standard deviations calculated in both dimensions over the 500 Monte Carlo diets are given by the white L shape near the lower-left corners of sufficiently large rectangles. Total resource demands of the plant based replacement diets as percentage of the corresponding demands of the replaced meat(s) are at the top of each panel, e.g., the mean all meat replacement plant diet uses 30 ± 2% of the cropland beef, poultry and pork currently jointly use (panel b).

Fig. S1c,d show the resources these leading items use, uniquely rearranged in descending order of importance for each of the four resources. (For the beef replacement, only four contributions are shown because those of the remaining items to the total resource uses are trivial.) There is some correspondence between items dominating by both mass and resource use (compare panel a and panels c in Fig. S1). Similarly, green peas, which dominate the mass of the beef replacement, claim substantial amounts of the 3 resources save land (Fig. S1d_1,4_). But exceptions to these expectations abound in individual burdens (e.g., the contribution of tofu to water needs of the all meat replacement). Similar but differently ordered information is recast in Fig. 3 to more finely resolve the composition of and overall resource use by the diets. The figure shows only items that dominate the mean resource use by the 500 randomized plant based diets replacing all meat (panel b–e) or beef alone (panels f–i). Once chosen based on their contribution to total resource use by the diet, we rank items by mass contributions (in g person^−1^ d^−1^, horizontal axes), with contributions to total resource use shown cumulatively along the vertical dimension. The items are identified in panel a, along with their contributions to the diets’ respective overall protein contents. In panels b–i, high resource users per g (e.g., water use by peanuts in the all meat replacement or Nr use by asparagus in the beef replacement) form tall rectangles, while low resource users per g (e.g., all resource use by soy) form flat, wide rectangles. While sharing some items (e.g., lentils or green peas), the two mean replacement diets also differ. These differences stem from nutritional differences between beef alone and the weighted mean of all meats, which enter the problem as the imposed bounds (the elements of **b**^(*a*)^ and **b**^(*b*)^ used in the respective optimizations, as explained in the Methods section).

To analyze this issue and better understand the composition of the replacement plant based diets, we identify inequality constraints that shape the solutions particularly strongly using the criteria described in the Methods section. For both beef and all meat replacements, constraints governing total mass and energy (associated with upper bounds), and monounsaturated fatty acid, vitamins D, B_3,6,12_, zinc, choline and selenium (associated with a lower bounds) prove critical.

Supplies of these critical nutrients are least likely to remain within desirable bounds following the considered meat-to-plant dietary shifts. Identifying such nutrients is thus an essential element in successfully, safely deploying animal-to-plant dietary shifts, to which the methodology introduced here is a powerful aid. It offers a practical message to those seeking plant based alternatives to meat in their diet: if increased caloric intake is undesired (because of weight, environmental or other concerns), supplies of protective monounsaturated fatty acids, Zn, Se, choline, and vitamins B_3_,_12_ are least likely to be adequately delivered by plant based alternative diets, and thus require special attention. Note, however, that while these nutrients are unlikely to be adequately supplied spontaneously (with no deliberate efforts) while minimizing resource use without increasing caloric intake, some relaxation of the minimization is enough to fully meet those requirements, as is clearly shown by the fact that each one of the 500 Monte Carlo (see Methods) diets fully meets the needs for most of these nutrients (but not B_12_) while also conforming with all other constraints (e.g., without appreciably increasing caloric intake relative to the lost calories in the forgone meat(s)). Ingesting enough of these nutrients thus requires deliberate efforts, but is eminently tractable.

It is also important to note that while the critical constraints are important to the composition of the solution diets Figs 1 and 3 present, they are not these diets’ sole final arbiters, for two reasons. First, the problem is high dimensional and features mostly inequalities, which together endow the solutions with considerable indeterminacy and flexibility. Second, if a solution exists (i.e., if a randomized problem proves feasible), the cost function also impacts the mass choices. To illuminate the tension between satisfying the nutritional constraints and resource use minimization in determining the solutions, we devise an index of suitability of plant items for satisfying the nutritional constraints, ${F}_{i}=\sum _{r}{\sigma }_{r}({a}_{ir}-{\bar{a}}_{r})/{s}_{r}$ The sum is over all constraints, and the sign parameter *σ*_*r*_ = 1 for lower bound constraints and −1 for upper bound ones. The parenthetical term is the deviation of *a*_*ir*_, the element of the nutritional composition coefficient matrix **A** (see Methods) corresponding to plant item *i* and nutrient *r*, from ${\bar{a}}_{r}$, the mean of the *r*th row (the mean content of nutrient *r* of all plant items considered). This deviation is then normalized and nondimensionalized by *s*_*r*_, the standard deviation of *r* content over all plant items. The logic behind the index is that obvious candidates for inclusion in the diet are plant items that are unusually rich in desirable nutrients (with lower bounds and *σ*_*r*_ = 1) but largely devoid of capped critical nutrients (with upper bounds and *σ*_*r*_ = −1). While no plant item is ideal, some—whose dimensionless *F*_*i*_ index is unusually high—are close. For such items, the ${a}_{ir}-{\bar{a}}_{r}$ terms are unusually positive for *σ*_*r*_ = 1 constraints, and unusually negative when *σ*_*r*_ = −1, both contributing to positive accumulation of the *F*_*i*_ sum, whose high positive values signify high compatibility of the item’s nutritional composition with the inequality nutritional constraints.

**Figure 4:**
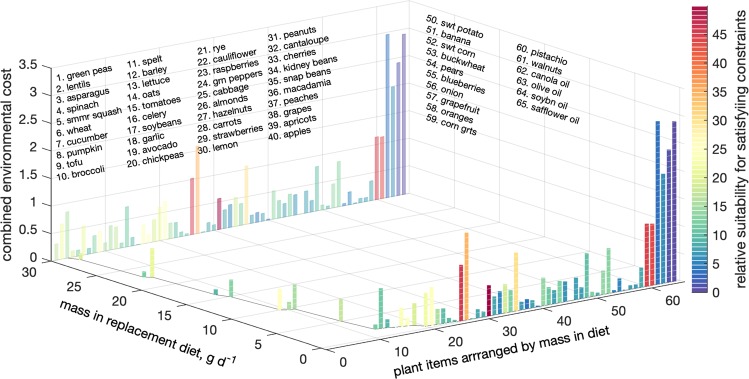
Analysis of the determinants of the beef replacement solution vector (the Supplementary Information offers the all meat counterpart to this plot). The plant items are arranged in descending order of mass prominence along the right horizontal axis (labeled “plant items arranged by mass in diet”), with the corresponding masses themselves shown along the left horizontal axis (labeled “mass in replacement diet, g d^−1^”). Individual food items are identified by the list of names on the z-axis that corresponds with descending order of mass (“plant items arranged by mass in diet”). The vertical bars show two attributes for each plant item. The bar heights show the combined nondimensional environmental cost (see Methods), with taller bars indicating higher resource use by the chosen mass of the item. To avoid parallax perception errors, white tickmarks show the rising environmental costs in increments of 0.05, and the full bars are projected in fainter colors on the back “wall” at mass = 30 g d^−1^. The bar colors, with a color scale shown on the right, show the relative compatibility of plant items with the critical constraints, with details in the subsection entitled Diet Composition, Nutrient Delivery, and share of Resource Use in the Results and Discussion section. The all-meat counterpart to this is given in the Supplementary Information file.

To show the suitability index in action for the all meat replacement, in Fig. 4 the items in the mean beef replacement diet are arranged in descending mass order as the solid curve at the zero environmental cost plane shows. For each item, the figure shows two additional attributes: the combined environmental cost as height against the vertical axis, and the dimensionless suitability index as a color evaluated against the color bar on the right. While noisy, the plot reveals three regimes. The high mass items are quite compatible with the inequalities *and* environmentally cheap. On the other (low mass) extreme (on the right) are plant items that are environmentally costly *and* less compatible with the inequalities. In between these two endmembers are plants whose environmental costs and nutritional compatibility are intermediate. For both replacements, this is quantified in Table 1.

**Table 1:** Three statistics of three groups of items in the mean solutions to the two replacement problems considered.

Mass Ranking of Items	Beef Replacement			All Meat Replacement		
	Mean Mass	Mean Environmental cost	Mean Suitability	Mean Mass	Mean Environmental cost	Mean Suitability
1 to 10	22	0.3	17	25	0.4	22
11 to 20	2.6	0.3	16	3.8	0.4	20
20 to 64	0.1	0.5	13	0.2	0.5	11

For each replacement diet, all items of the mean solution (over all Monte Carlo realizations) are arranged in descending order of mass, and then combined into the shown three item groups (the table**’**s rows).

**Table 2:** The 44 nutritional attributes addressed in the replacement calculations. “U”, “L” and “E” indicate attributes subject to upper bounds, lower bounds, and equality (only protein) respectively. Vit. = vitamin; FA = fatty acids; unsat. = unsaturated.

Item		Item		Item		Item	
kilocalories	U	Sodium	U	Folate	L	vit. K	L
protein	E	zinc	L	choline	L	sat. FA	U
fat	U	copper	L	B_12_	L	monounsat. FA	L
carbohydrates	U	manganese	L	vit. A	L	polyunsat. FA	L
total fiber	L	selenium	L	α carot.	L	phytosterols	L
sugar	U	vit. C	L	β carot.	L	cholesterol	U
calcium	L	thiamin	L	β crypt.	L	flavonoid	L
iron	L	riboflavin	L	lycopene	L	soluble fiber	L
magnesium	L	niacin	L	lut. + zea.	L	Ω_3_	L
phosphorus	L	panto. acid	L	vit. E	L	Ω_6_	L
potassium	L	vit. B_6_	L	vit. D	L	mass	U

The table’s suitability columns show that compatibility with the constraints is the primary determinant of the solution composition for both replacements; leading terms are highly compatible with the constraints (as indicated by their high mean *F*_*i*_ values), and trailing ones (rows 2 and 3) less so. This stems from the nontrivial challenges the replacements pose. The initial and decisive test for any linear programming problem is feasibility; not a specific combination of plant item masses, but confirmation that *a* combination of the considered items (here a different set for each Monte Carlo realization) can satisfy all nutritional constraints with individual item-specific masses below the imposed maxima. This primacy of compatibility explains the shown declining *F*_*i*_ values down the rows. Once compatibility is established, the emergent null space (non-uniqueness of the solution) presents the opportunity to minimize environmental costs by considering the resource needs of individual plant item members of the feasible set. This explains the similar mean costs of items 1–10 and 11–20, because the dominance of all 20 is governed primarily by feasibility, and only secondarily by cost minimization. Indirectly, it also explains the higher environmental costs of the trailing third group, whose items are on average simultaneously less compatible with the constraints and more environmentally costly. Their representative masses reflect cost minimization in the presence of enforced diversity due to the randomized specified minimum masses (see Methods). This is readily demonstrated by fitting a model of the form *c*_*i*_ = *c*_0_*e*^*αi*^ (where *c*_0_ is an optimizable parameter and, conformal with the Methods section, *c*_*i*_ denotes the combined nondimensional environmental cost of food item *i*, *α* is a fit parameter, and items are arranged according to the corresponding items’ masses with mass_*i*_ ≥ mass_*i*+1_ and *i* ∈ [21, 64]). These *α* s are positive for both the all meat and beef replacements, and significant at <0.008 and <0.030 respectively, showing that, as argued above, items’ environmental costs rise exponentially with their decreasing mass contributions. Critical inequality constraints, other more easily satisfied constraints, and item specific nutritional composition and resource use thus jointly determine the composition of each replacement diet and these diets’ statistics shown in Figs 1–3.

Returning briefly to Fig. 3, it also visualizes the disconnect between individual plant items’ chosen masses, fractional protein contributions, and resource use. For example, with ≈69 g person^-1^ d^-1^, soy and tofu jointly dominate the mean all meat replacement diet (first suitability group), delivering a full third of the total protein, yet account for about 12% of Nr and water needs, and <22% of the cropland needs. Similarly, lentils contribute the most protein to the beef replacing diet (about 3 g d^-1^ or 24%), but accounts for only 6% of this diet’s overall N fertilizer needs. Still in the beef replacement diet, by contrast, pumpkin delivers 6% of the mass but under 2% of the total protein while requiring ≈10% of the water and emissions (third suitability group).

These disconnects are not surprising—no obvious interdependence couples an item’s mass, protein delivery, and water or Nr needs per g—but are important because they highlight the potential for further resource savings by dietary choices beyond replacing meat achievable by favoring some plant items over others. The opposite side of this coin—the possibility of nutritionally or environmentally questionable plant based diets that nonetheless require considerable efforts to switch to—is equally important. Thus while the means of the plant and animal based categories differ markedly, the variability of individual items within each group renders the broad distinction—relatively desirable plant based food and relatively undesirable animal based group—an imperfect, and sometimes unhelpfully truncated approximation for the design of specific individual diets.

Notwithstanding our potentially hard to overcome culinary partiality toward animal protein, using the above solution diets to replace all meat or beef alone in the U.S. diet is thus possible^32^ through readily available plant combinations that offer diverse and nutritionally sound diets (Fig. 3). If required to fully replace the nutrients the replaced meats currently deliver, these replacement diets afford considerable individual land and fertilizer use and GHG emission savings, and stand to eliminate one third to one half of the total national dietary use of premium cropland, GHG emissions, and N fertilizer while increasing dietary water use by 15%. These conclusions are consistent with earlier, more general analyses^33,34^. Shifting from meats to plant based alternatives stands to also enhance significantly the population mean intake of such nutritionally protective substances as key phytonutrients^35^, minerals and vitamins, and total and soluble fiber^36^, but may also undermine intake of other protective nutrients, primarily monounsaturated fatty acids^37^, selenium, zinc^38^, and vitamin B_12_^26^. While these deficiencies are potentially clinically very serious, population data show that mostly or exclusively plant eating populations for the most part avoid such adverse consequences and enjoy extended health- and lifespans^17,26,37,39^.

## Methods

### Statement of the problem

We devise 500 Monte Carlo plant based diets that replace separately all meat (approximately 460 kcal, 30 g protein, and 170 g total mass per person per day) or beef alone (190 kcal, 12 g protein, and 66 g total mass person^−1^ d^−1^) in the mean American diet (MAD). We denote individual plant based solution diets generically **x**, or **x**^(*a*)^ and **x**^(*b*)^ when distinction between replacing all meat or beef alone is needed. Each replacement diet ${\bf{x}}\in {{\mathbb{R}}}^{35}$ comprises daily masses of 35 distinct plant items randomly drawn from a full pool of 64 plant items. The rather arbitrary choice of 35 items per MC realization strives to represent the typical natural day-to-day variability of actual diets, but repeating a subset of the calculations with 25 and 50 items per MC realization made no appreciable difference. The 500 member MC set is of course a miniscule fraction of the roughly 10^18^ possible draws of 35 items out of a pool of 64. This truncation is partly a necessity. Because the search for feasible solutions is computationally intensive, 500 is roughly the upper edge of tractability. Far more important to rendering 500 sufficient, however, is the small compositional variability feasible solutions exhibit (see Figs 3 and S1 of this paper). Each plant based solution diet consists of the masses of the 35 randomly chosen plant items (the elements of **x**) out of the full set of 64 described in the following section. Together, the 35 masses satisfy all nutritional constraints while minimizing resource use.

The nutritional values are held in the coefficient matrix **A**, whose element *a*_*ij*_ holds the content of nutritional attribute *j* in food item *i*. The column of **A** corresponding to rye, e.g., holds kcal (g rye)^−1^, g protein (g rye)^−1^, and all other considered nutritional attributes per g rye. Likewise, the row of **A** corresponding to vitamin K, say, holds the vitamin K content per gram of each food item considered.

### Nutritional data sources

Our nutritional data sources are based on earlier compiled dataset^5,14^ from USDA’s food composition tables and updated for certain nutrients. These include phytosterol^40^, omega 3 fatty acids^41,42^, flavonoids^43^ and soluble fiber. Despite our vigorous attempts, we are unable to obtain some nutritional attributes for some food items (grey cells in SI spreadsheet). The SI spreadsheet includes detailed references and comments for further information.

### Solution method

Using IBM^©^’s robust industry standard *cplexlp* linear programming implementation, we derive diets (obtain **x** vectors) that satisfy the nutritional constraints described below while minimizing **x**^T^**c**, the inner product of the solution vector **x** and a combined nondimensional cost function **c**, both ${{\mathbb{R}}}^{{N}_{p}}$ vectors, where the number of plant items is *N*_*p*_ = 35 for MC replacement plant diets and 63 for the statistics of all 500 replacement plant diets.

### Resource use information

The dimensional resource use values *c*_*ij*_ for cropland and Nr were derived from previous studies^5,12,14^, addressing production related resource use adjusted for loss^18^ (i.e. actual consumption, taking note of both food loss from farm to table and non-edible shares).

GHG emission information is based on life cycle assessments (LCAs) cited in refs^5,16^, using the 5^th^ Assessment Report^40^ of the Intergovernmental Panel on Climate Change^40^ one century Global Warming Potentials (GWP_100_) and converting retail weight to “loss adjusted”. While earlier work^44^ highlighted the oversimplifications of GWP_100_, it is the only widely used emission metric in the environmental life cycle assessment literature we use to obtain emissions per g of food product, and it facilitates comparison of our results with most earlier analyses. We do not address sequestration or emissions associated with land use changes.

Absent an LCA of a specific food item, we use similar items (e.g., using wheat values for spelt) or relevant categorical values^16^ (e.g. ‘nuts’ for almonds, see SI spreadsheet file). Augmenting earlier results, here we also consider fresh (“blue”) water usage taken from a meta-analysis of global food LCAs^16^ and corrected for loss. The animal food items’ resource usage and GHG emissions are derived from earlier U.S. studies^5,14^.

Our resource use and environmental impact data (see SI spreadsheet file) thus partly rely on available LCAs that sample imperfectly variability in space, time, and agro-practices, and may therefore not be nationally representative. While currently state of the art, our analysis is therefore somewhat tentative, awaiting further data availability and better characterization of the U.S. food system as a whole.

### Resource use cost function

Generalizing earlier^12–14,45^ single objective calculations, the *j*th element of the combined nondimensional cost function **c** (corresponding to the *j*th food item) is ${c}_{j}={\sum }_{i=1}^{4}{w}_{i}\,({c}_{ij}-\,{\bar{c}}_{i})/{s}_{i}.$ Here *i* = [1, 4] is the resource index corresponding to cropland, Nr, GHG emissions and water; *c*_*ij*_ is the use of the *i*th resource (land, reactive nitrogen or water use, or GHG emissions) by food item *j*; ${\bar{c}}_{i}\,$ is the mean over all food items of the *i*th resource use, ${\bar{c}}_{i}={64}^{-1}{\sum }_{j=1}^{64}{c}_{ij}$; and *s*_*i*_ is the corresponding sample standard deviation. The combined environmental cost is thus a weighted sum^46^ of the four dimensionless, scaled individual environmental costs, where the *i*th weight ${w}_{i}={c}_{i}^{a}/{c}_{i}^{n}$ is the fraction of the national total annual use of resource *i* (superscript *n*) that is attributable to livestock production (superscript *a*). For example, because roughly 150 million cropland acres are used for growing livestock feed^5,20^, which is ≈37% the total national cropland use of roughly 0.4 billion acres^47^, *w*_land_ ≈ 0.37. Similar considerations lead to *w*_*i*_ ≈ 0.21, 0.06, and 0.12 for *i* = [2, 4] corresponding to Nr^48^, GHG emissions^49^, and water^50^.

Like such predecessors as the Eco-Indicator^51^ or the safe operating approach to weighting life cycle impacts^52^, our choice^46^—minimizing the combined cost **c**—recognizes the multifaceted nature of environmental impacts of human activities. This multidimensionality dictates that a defensible unification of various impacts must guide environmental optimizations. Any such unification method must comparatively weigh various, potentially antithetical environmental impacts of the addressed human activity, likely with distinct mechanisms and physical units, and condense them into a single measure of combined environmental impact. This can take numerous nonunique forms, of which one is our choice of **c**, combining the disparate impacts into a single scalar cost function to be optimized. The precise choice of combination formalism and its parameter values are similarly nonunique. Our choice—weighted linear combination of relative burdens, with weights reflecting the dominance of livestock production over the current total national use of each resource—strives to economize environmental efforts and good will. With both limited, environmentally motivated recommendations or laws must only be enacted if they can be reasonably expected to improve nutrition while minimizing, or at least reducing environmental costs of food production. This quest for balanced environmental improvements (considering various environmental dimensions) underlies the combination of individual burdens the above **c** achieves. Yet the specific method of combining these burdens, whose physical units and societal significance vary widely, into a unified measure of the clearly subjective “environmental betterment” is neither unique nor straightforward.

The choice we make here, the above formulation of the weights *w*_*i*_, reflects our view that the most realistic expectation of suggested changes is not of a wholesale redesigning of the food system, but of incremental changes relative to today’s state. As such, at least in its inception, the envisioned change is reasonably viewed as a small perturbation to the current state. With this view, it makes sense to judge the societal desirability of a given small change in the use of a given resource—1% of today’s fertilizer application, say—partly by the importance of livestock to the overall national burden. Thus, e.g., efforts to reduce livestock related GHG emissions—which account for 6% of total national emissions—may arguably be secondary to efforts to reduce nationwide water pollution by eutrophication due to nitrogenous fertilizer runoff, of which about one fifth is due to livestock. (These values reflect the values of the GHG and Nr weights given earlier, and these considerations may well be further impacted by subjective choices that, e.g., single out a particular physical environmental burden as disproportionately important, thus potentially “vetoing” the above weighing).

### Nutritional constraints, redundancy

The cost function is minimized subject to nutritional constraints^19^. We do not use all nutritional attributes the USDA data^19^ address. We discard some (e.g., caffeine, trans fats) neither of the plant or meat items we consider contains. We also exclude from the calculations the constraints addressing carbohydrates and sugar, of which the replaced meats deliver essentially none, and B_12_ and cholesterol, of which plants deliver extremely little^53^ or none respectively; the notion of “replacement” is simply inapplicable for those nutrients. No replacement diet thus supplies any B_12_, a well known limitation of plant based diets^54^ readily ameliorated by supplements^24^, and all replacement diets contain more carbohydrates than the diets they replace. While this excess may be invoked by some^55^ as a nutritional liability of the plant based replacement diets, this is readily countered by the fact that all plant items in these diets are whole, complex, and minimally processed^56,57^.

Before using the remaining attributes, we test the corresponding inequality constraints for redundency^58^, as follows. Consider a lower bound constraint ${{\bf{a}}}_{i}^{T}{\bf{x}}\ge {b}_{i}$ ensuring that the inner product of **A**’s *i*th row and the sought solution vector of plant item masses—the sum $\mathop{\sum }\limits_{j=1}^{{N}_{p}}{a}_{ij}{x}_{j}\,\equiv {\hat{b}}_{i}$ of nutritional attribute *i* diet **x** delivers—does not fall below the *i*th imposed bound *b*_*i*_. The constraint is redundant if ${{\bf{a}}}_{i}^{T}{\bf{l}}\equiv {L}_{{\boldsymbol{i}}}\, > {b}_{i}$, where the *j* th element of **l** is the mass lower bound imposed on plant item *j*, satisfying *l*_*j*_ ≤ *x*_*j*_. Conversely, the upper bound constraint ${{\bf{a}}}_{i}^{T}{\bf{x}}\le {b}_{i}$ is redundant when ${{\bf{a}}}_{i}^{T}{\bf{u}}\equiv {U}_{{\boldsymbol{i}}} < {b}_{i}$. In both cases, even when all plant item masses attain their extreme values least favorable to feasibility, the inequalities are still identically satisfied, thus not constraining the solution.

We test all remaining constraints for redundancy by devising **U** = **Au** and **L** = **Al** (the vectors holding *L*_***i***_ and *U*_***i***_ for all considered nutritional attributes/constraints) from 500 randomly drawn 35 plant item sets, and calculating the percent of cases in these ensembles a given constraint proves redundant in the all meat or beef replacements. Some attributes (e.g., total and soluble fiber or lycopene) are entirely redundant for both replacements, reflecting these nutrients’ absence from meat (which means that the base value of their corresponding *b*_*i*_ is zero, so that any combination of nonnegative *x*_*i*_ will exceed the lower bound, thus identically satisfying the inequality). Of the full set of nutritional attributes in Table 2 and apart from the excluded carbohydrates, sugar, B_12_ and cholesterol, the following are not uniformly redundant in at least one of the two replacements: energy, protein, total fat, calcium, iron, magnesium, phosphorus, potassium, sodium, zinc, copper, selenium, vitamins A, E, C, D, K, B_1,2,3,5,6_, folate, choline, saturated fat, Ω_3,6_, poly-, and mono-unsaturated fatty acids, and mass, a total of 30 active constraints.

These 30 attributes define the reduced coefficient matrix ${{\bf{A}}}_{{\rm{r}}}\subset {\bf{A}}\in {{\mathbb{R}}}^{30\times 64}$, a row-wise subset of the full coefficient matrix ${\bf{A}}\in {{\mathbb{R}}}^{44\times 64}$ whose element *a*_*ij*_ is the amount of nutrient *i* in a g of plant item *j*, with *i* ∈ [1, 30] and *j* ∈ [1, 64]. When **A** premultiplies the solution vector **x**, the resultant vector ${\bf{A}}{\bf{x}}=\hat{{\bf{b}}}\in {{\mathbb{R}}}^{44}$ contains the predicted (hence the hat) daily delivery of all nutritional attributes by the diet whose composition the elements of **x** reflect. Of those, 30 attributes formally constrain **x**, while the remaining 14 are only evaluated after the solution **x** is obtained.

The 30 solution constraining attributes define what the individual plant item masses must jointly deliver, with minimum or maximum delivery bounds generally mirroring the forgone delivery by the replaced meat(s), denoted for the *i*th nutritional attribute (constraint) by ${b}_{i}^{(a)}$ and ${b}_{i}^{(b)}$ for all meat and beef, respectively. For *i* corresponding to fat, e.g., ${b}_{{\rm{fat}}}^{(a,b)}$ hold observed^18^ daily g fat contributed by all meat and by beef alone to the mean American diet, with ${\hat{b}}_{{\rm{fat}}}\le {b}_{{\rm{fat}}}^{(a,b)}$ holding.

Protein is the only equality; plant based alternative diets exactly replace the forgone meat protein. This reflects the fact that interpreting the environmental and nutritional consequences of dietary shift from meat to plant based diets is difficult unless the replaced meat protein is identically conserved by plant protein the replacement diets deliver. The protein conserving formulation does not disregard protein overconsumption in the U.S.^18^ or the suggestion that on average, plant eaters overconsume less protein than the full population^59^. Rather, this choice yields “per unit replaced meat protein” results that can be generalized to plant replacement of other meat protein consumption rate of interest.

The 29 active inequality constraints specify that all non-redundant nutritional attributes remain above or below their empirically derived imposed bounds, with the sense (≤ or ≥) of the inequalities given in Table 2 (as L or U respectively).

### Bound relaxations

The following few exceptions of imposed inequality constraint bounds proved necessary for ameliorating otherwise infeasible problems. In replacing all meat, we reduce the lower bounds of selenium, choline, and monounsaturated fatty acids from the full sum of deliveries by the three replaced meats to half these values, and raise permissible mass to 250 g (addressing the inevitable and largely positive bulky nature of plant based diets^60^). In replacing beef alone, we similarly relax selenium, choline, and monounsaturated fatty acids bounds, also reduce to 50% that corresponding to niacin (B_3_), and increase the mass upper bound to 300 g.

In addition to constraining overall mass, we also impose randomized lower and upper bounds on individual item masses, respectively *l*_*j*_ = max[0, *N*(0, 0.5)] and *u*_*j*_ = 20 ± *N*(0, 5) g for *j* = {all plant items}, where *N*(0, *s*) denotes random draws from a centered normal distribution with variance *s*^2^ for each Monte Carlo realization. Because garlic tends to feature in feasible solutions in levels (g garlic person^−1^ d^−1^) that exceed most people’s preferences, we set *u*_garlic_ = 5 ± *N*(0, 1)g.

### Identifying uniquely impactful constraints

To better understand the results and gain mechanistic insights into the composition of the replacement plant based diets, we seek to identify inequality constraints that impact the solution composition particularly assertively using two partially redundant criteria. The first characteristic of constraints that strongly control the solution is that each of the 500 found feasible MC solutions delivers an amount of nutrient *i* just barely distinct from the respective bound, trivially larger than an imposed lower bound or trivially smaller than an imposed upper bound. Like a teenager arriving home at 11:59 to just beat a “no later than midnight” curfew, such inequalities are thus effectively equalities in that all MC realizations satisfy for both *a* and *b* the condition $|{\hat{b}}_{ik}^{(a,b)}-{b}_{ik}^{(a,b)}| \lll {b}_{ik}^{(a,b)}$ so that the amount of nutritional attribute *i* the *k*th MC solution delivers is only immaterially distinct from the respective imposed bound. The defining characteristic of such constraints is that they satisfy $|\overline{{\hat{b}}_{ik}^{(a,b)}}-{b}_{ik}^{(a,b)}|/{b}_{ik}^{(a,b)}\,$ < 0.05 where the overbar denotes mean over the *k* = [1,500] MC realizations, namely that the mean absolute difference between predicted left hand side and the imposed right hand side is under 5% of the imposed one. A related characteristic of such outstandingly impactful constraints is a near zero variability of the reproduced left hand side term ${\hat{b}}_{ik}^{(a,b)}$, because all such terms are essentially equal to the corresponding imposed bound ${b}_{ik}^{(a,b)}$. This means that $\sqrt{{{\rm{var}}}_{k}({\hat{b}}_{ik}^{(a,b)})\,{[\overline{({\hat{b}}_{ik}^{(a,b)}2)}]}^{-1}}$ < 0.05, or essentially that the ratio of the variability of the predicted left hand sides to their mean (where both variance and mean are calculated over all MC realizations *k*) is small.

### Normalization of nutrient delivery in Figure 1

The main panel of Fig. 1 presents dimensional delivery of 16 nutrients by the two replaced and two replacement diets. To put these delivery values in perspective, and place them on equal footing, we normalize them element-wise by the truncated mean American diet (tMAD), **b**^(*t*)^ = A**x**^**MAD**^, the delivery of the 34 nutritional attributes by all 73 plant and animal items in our data set at the masses **x**^**MAD**^ they contribute to the mean American diet (MAD). Thus the ≈25% values for *β* carotene in Fig. 1a, e.g., means that the mean *β* carotene delivery by the two sets of 500 MC plant based replacement diets (replacing beef and all meat) is about 25% of the total delivery by **x**^**MAD**^.

## Supplementary information

Supplementary information file

Supplementary Dataset 1

## Data Availability

The original code used to generate the results of this study can be accessed at https://github.com/geshel/SciRepAug2019.git or at https://zenodo.org/badge/latestdoi/205003590.

## References

[1] Rockström J (2017). Sustainable intensification of agriculture for human prosperity and global sustainability. Ambio.

[2] Vanwalleghem T (2017). Impact of historical land use and soil management change on soil erosion and agricultural sustainability during the Anthropocene. Anthropocene.

[3] Davis KF (2015). Historical trade-offs of livestock’s environmental impacts. Environ. Res. Lett..

[4] Sakadevan, K. & Nguyen, M.-L. *Chapter Four - Livestock Production and Its Impact on Nutrient Pollution and Greenhouse Gas Emissions*. In (ed. Sparks, D. L.) **141**, 147–184 (Academic Press, 2017).

[5] Eshel, G., Shepon, A., Makov, T. & Milo, R. Land, irrigation water, greenhouse gas, and reactive nitrogen burdens of meat, eggs, and dairy production in the United States. *Proc. Natl. Acad. Sci. USA* **111** (2014). 10.1073/pnas.1402183111 PMC4143028 25049416

[6] De Ron A, Sparvoli F, Pueyo J, Bazile D (2017). Editorial: Protein Crops: Food and Feed for the Future. Front. Plant Sci..

[7] Song G, Li M, Fullana-i-Palmer P, Williamson D, Wang Y (2017). Dietary changes to mitigate climate change and benefit public health in China. Sci. Total Environ..

[8] Liu J (2015). Systems integration for global sustainability. Science (80-.)..

[9] Pelletier N, Tyedmers P (2010). Forecasting potential global environmental costs of livestock production 2000–2050. Proc. Natl. Acad. Sci. USA.

[10] Clark M, Tilman D (2017). Comparative analysis of environmental impacts of agricultural production systems, agricultural input efficiency, and food choice. Environ. Res. Lett..

[11] Tilman D, Clark M (2014). Global diets link environmental sustainability and human health. Nature.

[12] Eshel G, Shepon A, Noor E, Milo R (2016). Environmentally Optimal, Nutritionally Aware Beef Replacement Plant-Based Diets. Environ. Sci. Technol..

[13] Shepon, A., Eshel, G., Noor, E. & Milo, R. The opportunity cost of animal based diets exceeds all food losses. *Proc. Natl. Acad. Sci. USA* **115** (2018). 10.1073/pnas.1713820115 PMC5899434 29581251

[14] Shepon A, Eshel G, Noor E, Milo R (2016). Energy and protein feed-to-food conversion efficiencies in the US and potential food security gains from dietary changes. Environ. Res. Lett..

[15] Tallentire CW, Mackenzie SG, Kyriazakis I (2018). Can novel ingredients replace soybeans and reduce the environmental burdens of European livestock systems in the future?. J. Clean. Prod..

[16] Poore J, Nemecek T (2018). Reducing food’s environmental impacts through producers and consumers. Science (80-.)..

[17] Jahn JL, Stampfer MJ, Willett WC (2015). Food, Health & the Environment: A Global Grand Challenge & Some Solutions. Daedalus.

[18] United States Dept. of Agriculture, E. R. S. Loss-Adjusted Per Capita Food Availability Data. (2015).

[19] United States Dept. of Agriculture, Agricultural Research Service, N. A. L. National Nutrient Database for Standard Reference, Available at: http://ndb.nal.usda.gov/. (Accessed: 2nd November 2018) (2016).

[20] Eshel G, Shepon A, Makov T, Milo R (2014). Partitioning United States’ Feed Consumption Among Livestock Categories For Improved Environmental Cost Assessments. J. Agric. Sci..

[21] House J (2016). Consumer acceptance of insect-based foods in the Netherlands: Academic and commercial implications. Appetite.

[22] Craig WJ, Mangels AR (2009). Position of the American Dietetic Association: vegetarian diets. J. Am. Diet. Assoc..

[23] Hu FB (2003). Plant-based foods and prevention of cardiovascular disease: an overview. Am. J. Clin. Nutr..

[24] Donaldson MS (2000). Metabolic vitamin B12 status on a mostly raw vegan diet with follow-up using tablets, nutritional yeast, or probiotic supplements. Ann. Nutr. Metab..

[25] Das, S. K., Karlsen, M. C., Blanchard, C. & Roberts, S. B. Vegan Diets. In *Clinical Guide to Popular Diets* 99–112 (CRC Press, 2018).

[26] Woo KS, Kwok TCY, Celermajer DS (2014). Vegan Diet, Subnormal Vitamin B-12 Status and Cardiovascular Health. Nutrients.

[27] Katz DL, Doughty KN, Geagan K, Jenkins DA, Gardner CD (2019). Perspective: The Public Health Case for Modernizing the Definition of Protein Quality. Adv. Nutr..

[28] Briske, D. D. *Rangeland Systems: Processes, Management and Challenges* (Springer, 2017).

[29] United States Dept. of Agriculture, N. A. S. S. QuickStats, Available at: http://quickstats.nass.usda.gov (2015).

[30] Asem-Hiablie S, Battagliese T, Stackhouse-Lawson KR, Alan Rotz C (2018). A life cycle assessment of the environmental impacts of a beef system in the USA. Int. J. Life Cycle Assess..

[31] Nickerson, C. & Borchers, A. United States Department of Agriculture, Economic Research Service - How Is Land Used. *Amber Waves*, Available at: http://www.ers.usda.gov/amber-waves/2012-march/data-feature-how-is-land-used.aspx#.Vo_EHtCPVUQ. (Accessed: 8th January 2016) (2012).

[32] Melina V, Craig W, Levin S (2016). Position of the Academy of Nutrition and Dietetics: Vegetarian Diets. J. Acad. Nutr. Diet..

[33] Hallström E, Carlsson-Kanyama A, Börjesson P (2015). Environmental impact of dietary change: A systematic review. Journal of Cleaner Production.

[34] Tom MS, Fischbeck PS, Hendrickson CT (2015). Energy use, blue water footprint, and greenhouse gas emissions for current food consumption patterns and dietary recommendations in the US. Environ. Syst. Decis..

[35] Taylor-Baer Marion, Herman Dena (2017). From Epidemiology to Epigenetics: Evidence for the Importance of Nutrition to Optimal Health Development Across the Life Course. Handbook of Life Course Health Development.

[36] De Vadder F (2014). Microbiota-generated metabolites promote metabolic benefits via gut-brain neural circuits. Cell.

[37] Appleby PN, Key TJ (2016). The long-term health of vegetarians and vegans. Proc. Nutr. Soc..

[38] Schüpbach R, Wegmüller R, Berguerand C, Bui M, Herter-Aeberli I (2017). Micronutrient status and intake in omnivores, vegetarians and vegans in Switzerland. Eur. J. Nutr..

[39] Benatar JRB, Stewart RA (2017). P5322Cardiometabolic risk factors and plasma fatty acids in vegans results of an observational study. Eur. Heart J..

[40] USDA. National Nutrient Database for Standard Reference 1 Software v.3.9.5.1_2019-01-29, phytosterols.

[41] Devore EE (2009). Dietary intake of fish and omega-3 fatty acids in relation to long-term dementia risk. Am. J. Clin. Nutr..

[42] Wang DD (2016). Association of Specific Dietary Fats With Total and Cause-Specific MortalityAssociation of Dietary Fats and Total and Cause-Specific MortalityAssociation of Dietary Fats and Total and Cause-Specific Mortality. JAMA Intern. Med..

[43] Seema Bhagwat, D. B. H. & Holden, J. M. *USDA Database for the Flavonoid Content of Selected Foods*. (2013).

[44] Pierrehumbert RT, Eshel G (2015). Climate impact of beef: an analysis considering multiple time scales and production methods without use of global warming potentials. Environ. Res. Lett..

[45] Wilson Nick, Nghiem Nhung, Ni Mhurchu Cliona, Eyles Helen, Baker Michael G., Blakely Tony (2013). Foods and Dietary Patterns That Are Healthy, Low-Cost, and Environmentally Sustainable: A Case Study of Optimization Modeling for New Zealand. PLoS ONE.

[46] Eshel G (2010). A geophysical foundation for alternative farm policy. Environ. Sci. Technol..

[47] Nickerson, C., Ebel, R., Borchers, A. & Carriazo, F. *Major Uses of Land in the United States, 2007*. (2011).

[48] Doering, O. C., Galloway, J. N., Theis, T. I. & Swackhamer, D. I. *Reactive Nitrogen in the United States: An Analysis of Inputs, Flows, Consequences and Management Options. A Report of the (EPA-SAB-11-013)* (2011).

[49] United States Environemental Protection Agency. *Inventory of U.S. Greenhouse Gas Emissions and Sinks: 1990–2016* (2018).

[50] Maupin, M. A. *et al*. Estimated Use of Water in the United States in 2010 Circular 1405. *US Geological Survey*. **1405** (2010).

[51] Goedkoop, M., Hofstetter, P., Müller-Wenk, R. & Spriemsma, R. LCA methodology: The Eco-Indicator 98 explained. *Int. J. Life Cycle Assess*. (1998).

[52] Tuomisto HL, Hodge ID, Riordan P, MacDonald DW (2012). Exploring a safe operating approach to weighting in life cycle impact assessment - A case study of organic, conventional and integrated farming systems. J. Clean. Prod..

[53] Watanabe F, Yabuta Y, Bito T, Teng F (2014). Vitamin B12-containing plant food sources for vegetarians. Nutrients.

[54] Pawlak R, Lester SE, Babatunde T (2014). The prevalence of cobalamin deficiency among vegetarians assessed by serum vitamin B12: A review of literature. Eur. J. Clin. Nutr..

[55] Dehghan M (2017). Associations of fats and carbohydrate intake with cardiovascular disease and mortality in 18 countries from five continents (PURE): a prospective cohort study. Lancet.

[56] Siri-Tarino PW, Chiu S, Bergeron N, Krauss RM (2015). Saturated Fats Versus Polyunsaturated Fats Versus Carbohydrates for Cardiovascular Disease Prevention and Treatment. Annu. Rev. Nutr..

[57] Sonnenburg JL, Bäckhed F (2016). Diet–microbiota interactions as moderators of human metabolism. Nature.

[58] Paulraj S, Sumathi P (2010). A Comparative Study of Redundant Constraints Identification Methods in Linear Programming Problems. Math. Probl. Eng..

[59] Orlich, M. J. & Fraser, G. E. Vegetarian diets in the Adventist Health Study 2: A review of initial published findings. In *American Journal of Clinical Nutrition* **100** (2014). 10.3945/ajcn.113.071233 PMC4144107 24898223

[60] Sanders TAB (1999). The nutritional adequacy of plant-based diets. Proc. Nutr. Soc..

