# Author Correction: Environmentally Optimal, Nutritionally Sound, Protein and Energy Conserving Plant Based Alternatives to U.S. Meat

**DOI:** 10.1038/s41598-019-50289-8

**Published:** 2019-09-20

**Authors:** Gidon Eshel, Paul Stainier, Alon Shepon, Akshay Swaminathan

**Affiliations:** 1 Physics Department, Bard College, Annandale-on-Hudson, NY 12504-5000 USA; 2 Harvard College, Cambridge, MA USA; 3 Department of Nutrition, T. H. Chan School of Public Health, Harvard University, Boston, USA

Correction to: *Scientific Reports* 10.1038/s41598-019-46590-1, published online 08 August 2019

This Article contained errors.

Immediately prior to publication the Authors discovered an error in the code used in the study in the naming of a plant food item. The authors replaced USDA plant item names, which are very long and contain too much information for graphical presentation, with shorter names. The authors used a Matlab cell array, in which items are indexed with values incremented by one for each added item. In the original code, one index in this item list was erroneously repeated, which meant that reported plant names were for items with an index off by one (for example, green peppers were listed instead of green peas which preceded them in the alphabetized list, pears replaced peanuts, and so on). This affects presentations of the data in Figure 3, 4, S1, and S2 as well the names of food items presented in the text.

In addressing this issue, the authors revisited all calculations. Because the results are derived using a Monte Carlo code whose randomization this time around is distinct from the original one, this resulted in minor differences in all other figures and in the numerical values reported in Table 1. These differences arose exclusively from being derived from two distinct randomizations.

The item naming errors in the paper are corrected and apart from the randomization-related differences have no effect on the environmental and nutritional improvements or on the paper’s conclusions.

In the Abstract:

“We develop a new methodology for identifying nutritional constraints whose satisfaction by plant eaters is challenging, disproportionately shaping the optimal diets, singling out energy, mass, monounsaturated fatty acids, vitamins B_3,12_ and D, choline, zinc, and selenium. By replacing meat with the devised plant alternatives—dominated by soy, green pepper, squash, buckwheat, and asparagus—Americans can collectively eliminate pastureland use while saving 35–50% of their diet related needs for cropland, Nr, and GHG emission, but increase their diet related irrigation needs by 15%.”

now reads:

“We develop a new methodology for identifying nutritional constraints whose satisfaction by plant eaters is challenging, disproportionately shaping the optimal diets, singling out energy, mass, monounsaturated fatty acids, vitamins B_3,6,12_ and D, choline, zinc, and selenium. By replacing meat with the devised plant alternatives—dominated by tofu, soybeans, peanuts, and lentils—Americans can collectively eliminate pastureland use while saving 35-50% of their diet related needs for cropland, Nr, and GHG emission, but increase their diet related irrigation needs by 15%.”

In the Results, in subsection ‘Diet Composition, Nutrient Delivery, and share of Resource Use’:

“In daily per capita mass, buckwheat, soy, pears, and kidney beans dominate the all meat replacement, while green pepper, soy, asparagus, and squash dominate the beef only replacement (Fig. S1a,b, which present the eight most dominant plant items in the mean replacement diets calculated over the 500 Monte Carlo realizations).”

now reads:

“In daily per capita mass, tofu, soybeans, peanuts, and lentils dominate the all meat replacement, while green peas, lentils, asparagus, and spinach dominate the beef only replacement (Fig. S1a,b, which present the eight most dominant plant items in the mean replacement diets calculated over the 500 Monte Carlo realizations).“

In this same subsection:

“Similarly, green peppers, which dominate the mass of the beef replacement, claim substantial amounts of the 3 resources save land (Fig. S1d_1,4_). But exceptions to these expectations abound in individual burdens (e.g., the contribution of buckwheat to water needs of the all meat replacement).”

now reads:

“Similarly, green peas, which dominate the mass of the beef replacement, claim substantial amounts of the 3 resources save land (Figs. S1d_1,4_). But exceptions to these expectations abound in individual burdens (e.g., the contribution of tofu to water needs of the all meat replacement).”

The section:

“In panels b-i, high resource users per g (e.g., water use by pears or Nr use by asparagus) form tall rectangles, while low resource users per g (e.g., all resource use by soy) form flat, wide rectangles. While sharing some items (e.g., soy or green peppers), the two mean replacement diets also differ.”

now reads:

“In panels b-i, high resource users per g (e.g., water use by peanuts in the all meat replacement or Nr use by asparagus in the beef replacement) form tall rectangles, while low resource users per g (e.g., all resource use by soy) form flat, wide rectangles. While sharing some items (e.g., lentils or green peas), the two mean replacement diets also differ.”

Additionally, as a result of re-randomisation, the value obtained for vitamin B_6_ now reaches the significance threshold, and therefore the section:

“For both beef and all meat replacements, constraints governing total mass and energy (associated with upper bounds), and monounsaturated fatty acid, vitamins D, B_3,12_, zinc, choline and selenium (associated with a lower bounds) prove critical.”

reads:

“For both beef and all meat replacements, constraints governing total mass and energy (associated with upper bounds), and monounsaturated fatty acid, vitamins D, B_3,6,12_, zinc, choline and selenium (associated with a lower bounds) prove critical.”

The results presented in Table 1 were also modified as a result of the new randomization. Table 1 in the Article was updated and the original table appears below as Table 1:

**Table 1:** .

mass ranking of items	beef replacement			all meat replacement		
	mean mass	mean environmental cost	mean suitability	mean mass	mean environmental cost	mean suitability
1 to 10	22	0.4	16	26	0.4	22
11 to 20	2.3	0.3	13	3.7	0.4	14
20 to 64	0.1	0.6	12	0.2	0.6	11

The paragraph:

“Returning briefly to Fig. 3, it also visualizes the disconnect between individual plant items’ chosen masses, fractional protein contributions, and resource use. For example, with ≈68 g person^−1^ d^−1^, buckwheat and tofu jointly dominate the mean all meat replacement diet (first suitability group), delivering a full third of the total protein, yet account for about 12% of Nr and water needs, and <22% of the cropland needs. Similarly, soy contributes the most protein to the beef replacing diet (about 3 g d^-1^ or 24%), but accounts for only 6% of this diet’s overall N fertilizer needs. Still in the beef replacement diet, by contrast, raspberries deliver 8% of the mass but under 2% of the total protein while requiring 12–14% of the water and emissions (third suitability group).”

now reads:

“Returning briefly to Fig. 3, it also visualizes the disconnect between individual plant items’ chosen masses, fractional protein contributions, and resource use. For example, with ≈69 g person^-1^ d^-1^, soy and tofu jointly dominate the mean all meat replacement diet (first suitability group), delivering a full third of the total protein, yet account for about 12% of Nr and water needs, and <22% of the cropland needs. Similarly, lentils contribute the most protein to the beef replacing diet (about 3 g d^-1^ or 24%), but accounts for only 6% of this diet’s overall N fertilizer needs. Still in the beef replacement diet, by contrast, pumpkin delivers 6% of the mass but under 2% of the total protein while requiring $\approx$ 10% of the water and emissions (third suitability group).”

Figures 1, 2, 3, and 4 are replaced in the Article with the correct versions; the original versions are reproduced below as Figure 1 through 4.

**Figure 1:**
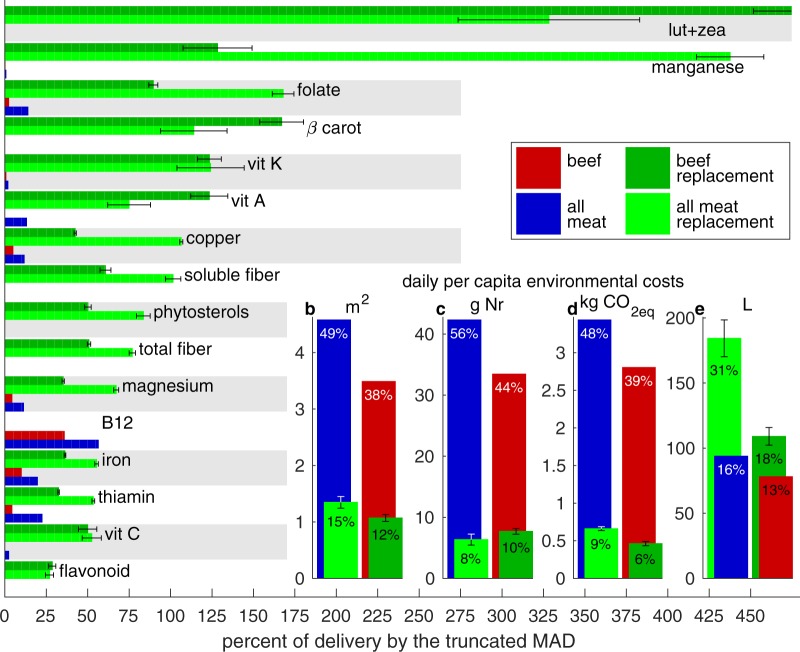
Original version of Figure 1, which is now replaced in the Article.

**Figure 2:**
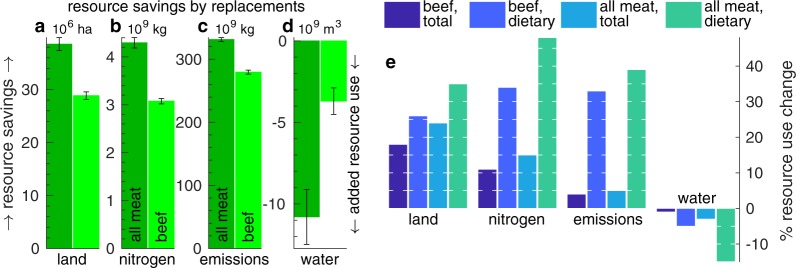
Original version of Figure 2, which is now replaced in the Article.

**Figure 3:**
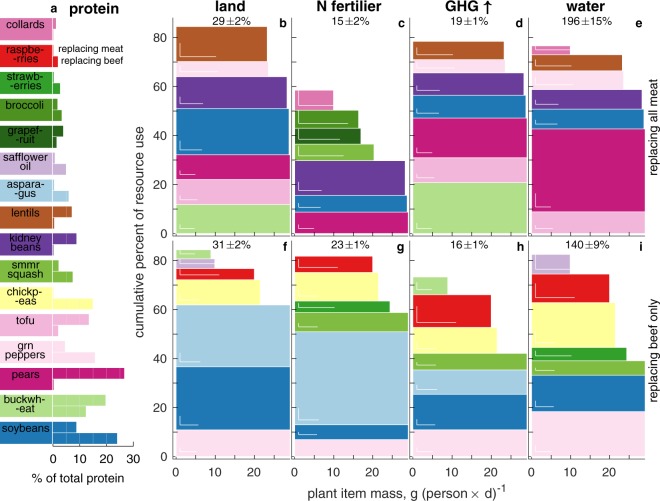
Original version of Figure 3, which is now replaced in the Article.

**Figure 4:**
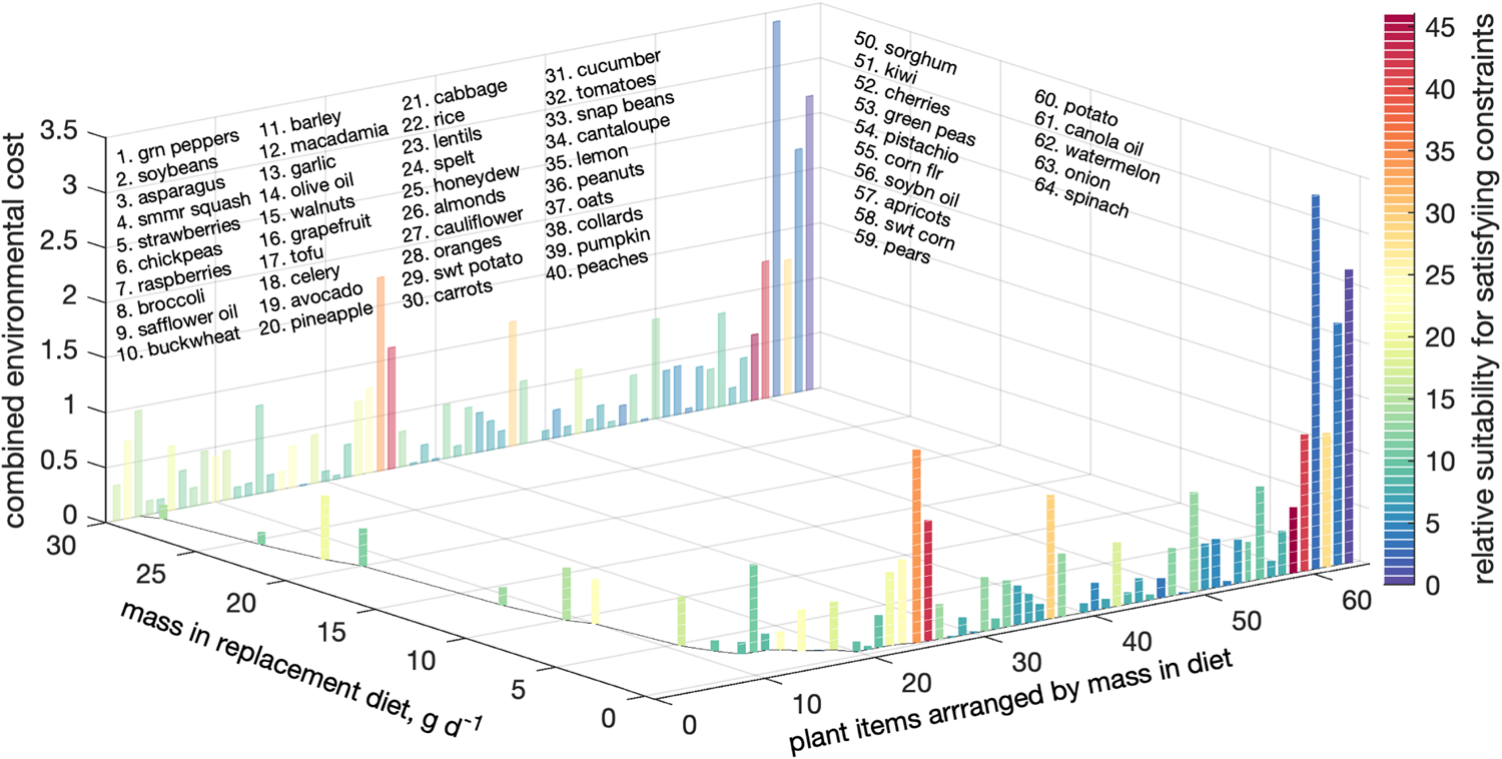
Original version of Figure 4, which is now replaced in the Article.

Figure 3 legend was also corrected. The numerical value of cropland usage is corrected as a result of re-randomization:

“For example, contributing ≈29 g cap.^−1^ d^−1^, summer squash is prominent (4^th^ by mass) in the beef replacement diet. Yet because it is responsible for under 1% of total land use, it is not a top-5 land user, and is thus absent from panel f. Standard deviations calculated in both dimensions over the 500 Monte Carlo diets are given by the white L shape near the lower-left corners of sufficiently large rectangles. Total resource demands of the plant based replacement diets as percentage of the corresponding demands of the replaced meat(s) are at the top of each panel, e.g., the mean all meat replacement plant diet uses 29 ± 2% of the cropland beef, poultry and pork currently jointly use (panel b).”

now reads:

“For example, contributing ≈29 g cap.^−1^ d^−1^, spinach is prominent (4^th^ by mass) in the beef replacement diet. Yet because it is not a top land user, it is thus absent from panel f. Standard deviations calculated in both dimensions over the 500 Monte Carlo diets are given by the white L shape near the lower-left corners of sufficiently large rectangles. Total resource demands of the plant based replacement diets as percentage of the corresponding demands of the replaced meat(s) are at the top of each panel, e.g., the mean all meat replacement plant diet uses 30 ± 2% of the cropland beef, poultry and pork currently jointly use (panel b).”

Finally, item names in Supplementary Figures 1 and 2 were also affected. The Supplementary Information was replaced with a version containing correct figures, but the original Figures S1 and S2 are reproduced below as Figure 5 and Figure 6.

**Figure 5:**
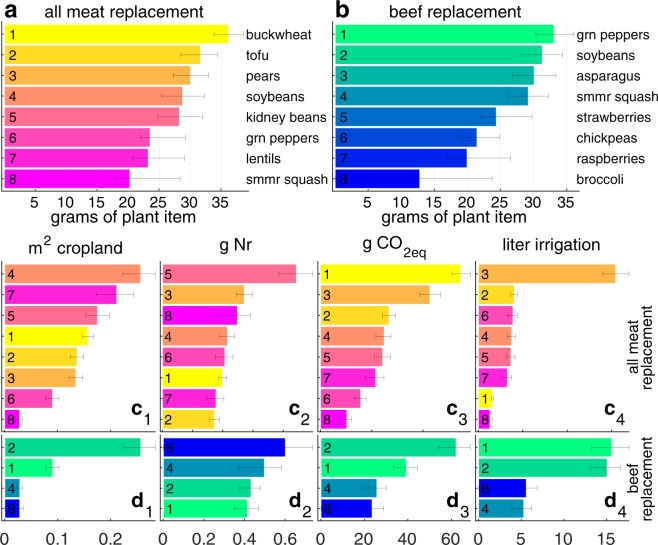
Original version of Supplementary Figure 1.

**Figure 6:**
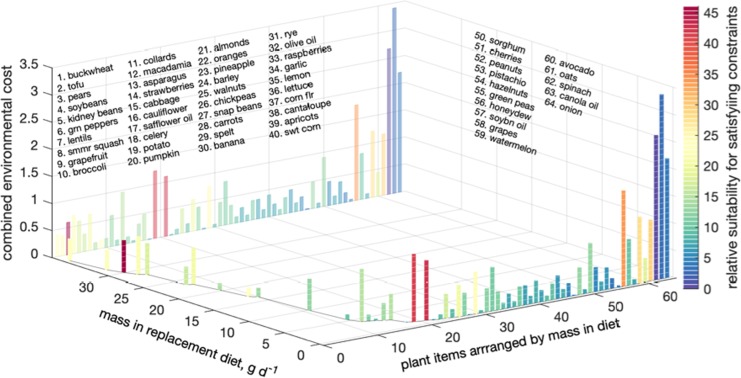
Original version of Supplementary Figure 2.

To aid the readers wishing to reproduce the results, the Article now includes a new Code Availability section that reads:

“Code Availability

The original code used to generate the results of this study can be accessed at https://github.com/geshel/SciRepAug2019.git or at https://zenodo.org/badge/latestdoi/205003590.”

These errors do not affect the main conclusions of the Article.

