# Supplementary material for: Environmentally Optimal, Nutritionally Sound, Protein and Energy Conserving Plant Based Alternatives to U.S. Meat
Source: Sci Rep. 2019 Aug 8;9:10345. doi: 10.1038/s41598-019-46590-1 (PMC6687707; doi:10.1038/s41598-019-46590-1)
Supplement: Supplementary file 1 — Supplementary information file [file 41598_2019_46590_MOESM1_ESM.docx]

**Supplementary Information for *Environmentally Optimal, Nutritionally Sound, Protein and Energy Conserving Plant Based Alternatives to U.S. Meat***

Gidon Eshel^1,*^, Paul Stainier^2^, Alon Shepon^3^, Akshay Swaminathan^2^

^1,^*^*^* Corresponding author. Physics Dept., Bard College, Annandale-on-Hudson NY 12504-5000 USA, [geshel@gmail.com](mailto:geshel@gmail.com), (413) 717-2187.

^2^Harvard College, Cambridge, MA, USA

^3^Department of Nutrition, Harvard T.H. Chan School of Public Health, Boston, MA, USA

**Supplementary Figure S1**

Figure S1: Replacement masses (in g person^-1^ d^-1^) of the eight mass dominating items in the plant based diets replacing all meat (a) or only beef (b) in the mean American diet. The rightward extent of each bar denotes the contribution in g d^-1^ of the corresponding plant item to the mean per capita replacement diet. The range whiskers show the central 50% of the full Monte Carlo distribution, excluding the 25% extreme realizations of either sign. Plant item specific environmental burdens associated with the all meat and beef only replacement diets are presented (in the shown units per person per day) in panels c_1-4_ and d_1-4_ respectively. Individual plant items are identified by color (with distinct color pallets for the all meat and beef replacements), and are also enumerated near the bars’ left edges. This permits identification of plant items that dominate the overall environmental burdens in panels c and d. For the beef replacement (panels d), only the shown four items have non-vanishing contributions to total burdens.

**Figure S2:** The all-meat analog of Fig. 4 of the main text. Analysis of the determinants of the all meat replacement solution vector. The plant items are arranged in descending order of mass prominence along the right horizontal axis (labeled “plant items arranged by mass in diet”), with the corresponding masses themselves shown along the left horizontal axis (labeled “mass in replacement diet, g d^-1^”). Individual items are identified by name in descending order of mass on the back “walls”. The vertical bars show two attributes for each plant item. The bar heights show the combined nondimensional environmental cost (see Methods), with taller bars indicating higher resource use by the chosen mass of the item. To avoid parallax perception errors, white tickmarks show the rising environmental costs in increments of 0.05, and the full bars are projected in fainter colors on the back “wall” at mass = 38 g d^-1^. The bar colors, with a color scale shown on the right, show the relative compatibility of plant items with the critical constraints (using the suitability index), with details in the subsection entitled Diet Composition, Nutrient Delivery, and share of Resource Use in the Results and Discussion section.
